# Biomimetic microenvironmental preconditioning enhance neuroprotective properties of human mesenchymal stem cells derived from Wharton's Jelly (WJ-MSCs)

**DOI:** 10.1038/s41598-020-74066-0

**Published:** 2020-10-09

**Authors:** Wioletta Lech, Anna Sarnowska, Zuzanna Kuczynska, Filip Dabrowski, Anna Figiel-Dabrowska, Krystyna Domanska-Janik, Leonora Buzanska, Marzena Zychowicz

**Affiliations:** 1 Department of Stem Cell Bioengineering, Mossakowski Medical Research Centre, Polish Academy of Sciences, 5 Pawinskiego Street, 02-106 Warsaw, Poland; 2 Translational Platform for Regenerative Medicine, Mossakowski Medical Research Centre, Polish Academy of Sciences, 5 Pawinskiego Street, 02-106 Warsaw, Poland; 3 1st Department of Obstetrics and Gynecology, Faculty of Medicine, Medical University of Warsaw, Starynkiewicza Square 1/3, 02-015 Warsaw, Poland

**Keywords:** Mesenchymal stem cells, Stem-cell niche, Biomaterials, Stem cells, Regeneration and repair in the nervous system

## Abstract

Tuning stem cells microenvironment in vitro may influence their regenerative properties. In this study Wharton's Jelly-derived mesenchymal stem cells (WJ-MSCs) were encapsulated in 3D hydrogels derived from human fibrin (FB) or platelet lysate (PL) and the oxygen level was adjusted to physiological normoxia (5% O_2_). The influence of the type of the scaffold and physiological normoxia conditions was tested on the WJ-MSCs' survivability, proliferation, migratory potential, the level of expression of selected trophic factors, cytokines, and neural markers. Encapsulated WJ-MSCs revealed high survivability, stable proliferation rate, and ability to migrate out of the hydrogel and the up-regulated expression of all tested factors, as well as the increased expression of neural differentiation markers. Physiological normoxia stimulated proliferation of encapsulated WJ-MSCs and significantly enhanced their neuronal, but not glial, differentiation. Ex vivo studies with indirect co-culture of organotypic hippocampal slices and cell-hydrogel bio-constructs revealed strong neuroprotective effect of WJ-MSCs against neuronal death in the CA1 region of the rat hippocampus. This effect was potentiated further by FB scaffolds under 5% O_2_ conditions. Our results indicating significant effect of oxygen and 3D cytoarchitecture suggest the urgent need for further optimization of the microenvironmental conditions to improve therapeutical competence of the WJ-MSCs population.

## Introduction

Tissue engineering and regenerative medicine are currently vast and rapidly growing research fields. The intrinsic qualities of stem cells, including differentiation potential and self-renewal, make them a frontline source for the cell therapy^1^. However, protection of the transplanted cells from the host immunological attack and the modulation of their properties by in vitro preconditioning are the main issues to consider for successful treatment^2,3^. Thus, the next important step toward regenerative personalized medicine would be formation of biologically active bio-constructs: scaffolds populated by stem cells that could replace damaged tissue and become actively involved in the immunological defense system.

Decades of in vitro and in vivo experiments have shed a new light on diverse characteristics and biochemical pathways involved in actions of stem cells derived from different sources and populations. It has been proven that even stem cells isolated from the same source can behave differently if cultured under differing conditions^4,5^. Thus, in order to obtain therapeutically competent cell populations standardization of the in vitro culture in regard to recipient tissue requirements is necessary^6^.

In pursuit of the ultimate goal of developing artificial tissues similar to native ones, several preconditioning factors of stem cell culture were performed, including: reduced oxygen level (physiological normoxia) instead of commonly used atmospheric level (21% O_2_), optimal stem cell density, specific growth media composition, 3D versus 2D culture with different types of 3D scaffold itself. Taking together, they warrant the need for further evaluation^5^.

Mesenchymal stem/stromal cells (MSCs), a mesodermal population that can be derived from both adult and birth tissues, are currently the type of stem cells most widely used in clinical trials (www.clinicaltrials.gov)^7^. Loss of potency, inconsistent quality, the invasive nature of procedures used for cell isolation and time needed to expand cell culture severely, limit the use of classical source of MSCs such as bone marrow for many clinical applications. MSCs isolated from birth tissue display similar general characteristics of MSCs derived from adult tissue but exhibits higher proliferative potential and fewer signs of senescence as compared to MSCs obtained from other sources^8^. Wharton's Jelly (WJ), being rich in perinatal MSCs, is acquired from umbilical cord tissue that is typically disposed of as medical waste. Therefore, harvesting these cells does not pose any threat to the donor and incurs minimal costs.

Despite low immunogenicity and strong immunomodulatory properties mesenchymal stem cells derived from Wharton's Jelly (WJ-MSCs) display high capacity to spontaneously differentiate towards a neural lineage^9^. However source of stem cells, their isolation method^4^ and subsequent transplantation, can significantly affect their properties.

Recently, our research team demonstrated that mesenchymal stem cells derived from human Wharton's Jelly secreted plethora anti-inflammatory, anti-apoptotic, angiogenic, and neurogenic factors^4,10^. Basing on this data, WJ-MSCs were chosen for this study to repopulate biocompatible scaffolds and, further, to evaluate their therapeutic values in changeable biomimetic conditions in vitro.

Biomedical research combining stem cells and scaffold engineering requires multidisciplinary approaches that include biology and biotechnology to control cell distribution and dynamics of cell culture, as well as chemistry and physics, to construct the scaffold of required composition and cytoarchitecture. An abundance of different modalities led to some confusion concerning scaffold types and generations. Therefore, a very recent effort was made to regulate this field and categorize scaffolds in a seven-layer format^11^. Ideal scaffold should be biocompatible, biodegradable, stable, flexible mechanically, easy and inexpensive in production, non-toxic, and providing dynamic interactions that foster and regulate stem cells in a way that mimics naturally occurring events in a cellular microenvironment^6,12^. Regarding the tissue-engineering point of view, the goal of a scaffold is to successfully mimic the biology of the extracellular matrix (ECM) by providing a 3D microenvironment suitable for cell adhesion and proliferation under specific chemical and biophysical stimuli^13^.

However, when transition of stem-cell therapy from lab to bedside is required, the standardized, large-scale propagation of clinically relevant MSCs in an animal-serum-free medium is an essential factor profoundly defining the overall safety of stem cell therapies^14^.

Two scaffold types were used to encapsulate WJ-MSCs for this study. One type consisted of hydrogel made from human fibrin (FB), the other from human platelet lysate (PL).

Fibrin is a serum-derived protein which plays a vital role in process of coagulation. In order to stop bleeding, thrombin cleaves fibrinogen into fibrin monomers that assemble into a fibrous 3D network with the physical properties of soft tissue^15^. Recent research indicates that 3D fibrin scaffolds function as an efficient material facilitating process of engineering tissues obtained from pluripotent stem cells^16^. Moreover, it was demonstrated that embedding of neural stem cells in 3D fibrin scaffolds increase cellular survival and their further neuronal differentiation after transplantation into the injured spinal cord^16^. Unfortunately, fibrins' rapid degradation remains a major limitation for many tissue-engineering applications, as was shown in the process of supporting differentiation of human pluripotent stem cells^17^. Nevertheless, a recent discovery proved that fibrin gel obtained from cord blood is characterized by a specific protein profile that contributes to tissue regeneration, suggesting further study is warranted^5^.

Platelet lysate is currently under deep investigation as an innovative compound in the field of tissue repair and regeneration. In addition to effectively support MSCs culture, PL-based scaffold networks have been proven to enhance fibroblasts and endothelial cells adhesion and proliferation^18^. Additional advantages of PL-based scaffolds include its human origin and its uniquely intrinsic immunomodulatory properties. Recent research suggests PL-rich scaffolds do not change expression of surface markers on cultured cells^18^ and they reduce culture time by increasing growth rate of the cells. This type of the scaffold has also been found to effectively support cellular multilineage differentiation capacity of stem cells, thereby encouraging development of osteoblasts, chondrocytes, adipocytes, neurons, astrocytes, and other cell types^19,20^. These properties may prove to be of great value when designing 3D scaffolds to replace large tissue fragments^21^.

One must consider both the immunological properties of stem cells and scaffold endurance when developing a scaffold for transplant. For best results, bioconstruction should closely mimic the natural microenvironment of the highly specialized tissue at the target zone. To do so, three basic issues must be addressed: type of stem cells, type of scaffold biomaterial, and the most appropriate culture method.

Therefore, to find out and evaluate in vitro microenvironment supporting Wharton's Jelly-derived mesenchymal stem cells regenerative parameters, three-dimensional (3D) scaffolds based on human-derived proteins (i.e., platelet lysate or human plasma fibrinogen) were combined with the oxygen level adjusted to "physiological normoxia" in a chamber that provides constant culture conditions (*Xvivo System*, Biospherix).

This study indicates that physiological normoxia and 3D microenvironmental conditions induce positive neuroprotective and immunomodulatory response of WJ-MSCs in injured neuronal tissue, which is significantly different as compared to the control, 2D conditions. Furthermore, it highlights an urgent need to develop appropriate protocols for culturing, phenotypic verification, and biosafety control of acquired cells, as well as the protocols for enhancing therapeutic properties of transplanted cells and survival after transplantation. The proposed models of scaffolds/cell hybrids show promise of future use in MSCs-based therapy.

## Results

### Influence of different microenvironments on WJ-MSCs viability, proliferation rate, and gene-expression profile

**Figure 1:**
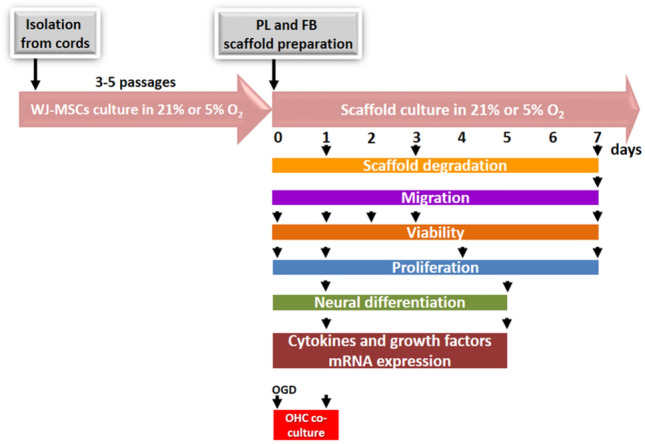
Experimental workflow. WJ-MSCs were cultured in 21% O_2_ and 5% O_2_ in a special chamber that provides constant oxygen level. After 3D scaffold preparation, WJ-MSCs encapsulated in the hydrogels were kept in the same culture conditions. Scaffold degradation, cell migration, viability, proliferation rate, neural differentiation, cytokine, and growth factors expression, as well as co-culture with organotypic hippocampal slices (OHC), were performed at different time points during 7 days of culture.

The WJ-MSCs were seeded in two types of hydrogels: scaffolds made from PL and from fibrinogen solution (5 mg/mL), both cross-linked with thrombin and cultured for up to 7 days in different (21% or 5%) oxygen culture conditions. Physiological normoxia conditions were achieved in a chamber set to maintain a constant level of O_2_, CO_2,_ and temperature throughout the entire culture. Physiological normoxia of 5% O_2_ was maintained even during the process of changing culture media. After 3D scaffold preparation, WJ-MSCs encapsulated in the hydrogels were kept in the same culture conditions (Fig. 1).

**Figure 2:**
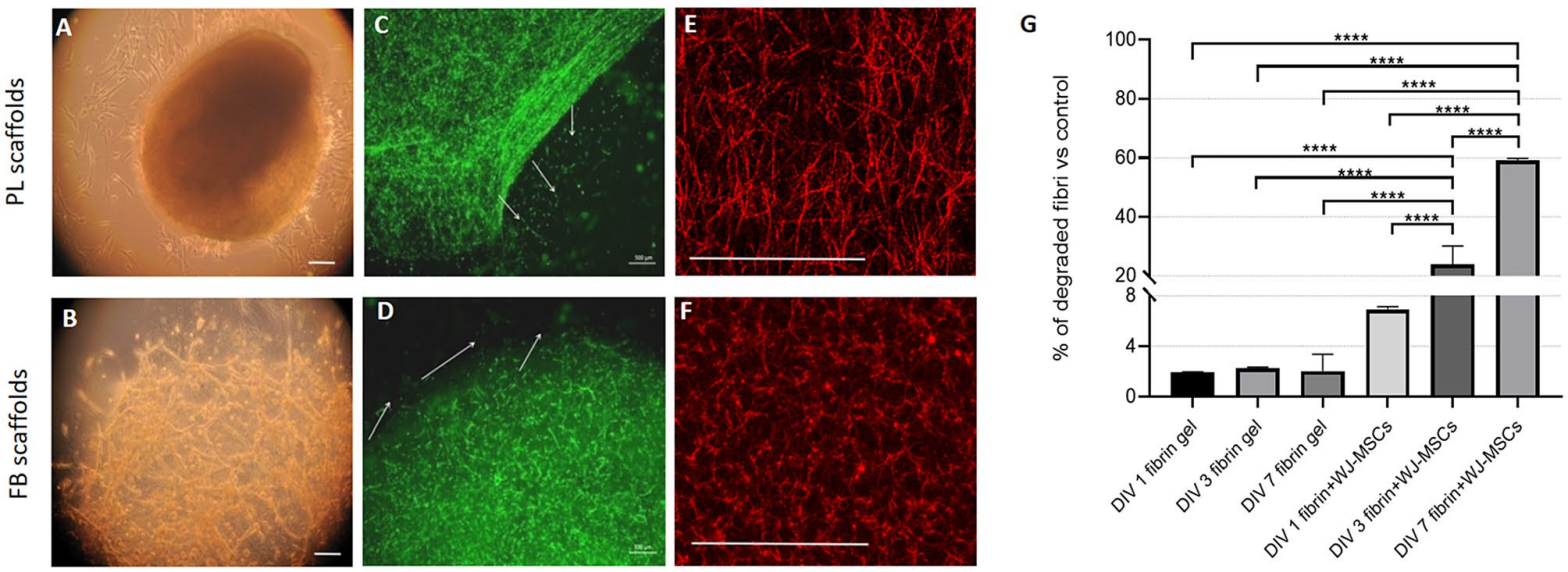
Scaffold morphology and characteristic. WJ-MSCs have been immersed in the hydrogels made from platelet lysate (**A**) or fibrinogen (**B**) and cultured for up to 7 days in vitro. Cells seeded in the scaffolds and labeled with CMFDA (green, **C**, **D**) were able to migrate outside the hydrogel (**C**, **D**, arrows). To visualize scaffold structure, the fibrin network was labeled by Alexa Fluor 546-conjugated human plasma fibrinogen (red, **E**, **F**). At certain time points (1, 3, 7 DIV) the FB scaffolds or FB with WJ-MSCs were analyzed for degradation rate. Calculation was based on the fluorescence of released labeled fibrinogen (% of fluorescence vs. fluorescence of totally degraded scaffold, ± SD, **G**). Abbreviations: PL- platelet lysate, FB—fibrin, DIV- days in vitro. Scale bars are presented as 250 µm (**A**, **B**), 500 µm (**C**, **D**) and 50 µm (**E**, **F**). Statistical analysis was performed using One Way ANOVA followed by Tukey’s multiple comparisons test, ****p < 0.0001.

Following entrapment within FB and PL scaffolds, WJ-MSCs were found well dispersed (Fig. 2A,B) and cells started to migrate outside the scaffolds (green cells labeled with CMFDA—5-chloromethylfluorescein diacetate, Fig. 2C,D). Using a fluorochrome-conjugated fibrinogen stain, it is possible to see formation of fibrin fibers in both hydrogel types. Thus, Alexa 546-conjugated fibrinogen was used to stain fibrin networks that were then cross-linked with thrombin, creating fibers of diverse diameter and random arrangement. The platelet lysate is composed of approximately 0.43 mg/mL of fibrinogen^22^, thus, after crosslinking with thrombin, scaffold structure became more fibrous and delicate and the fibrin fibers were longer and thinner (600 nm in diameter, data not shown). Fibrinogen at a concentration of 5 mg/mL cross-linked with 2 U/mL of thrombin gave rise to spongy structure, with the fibers of 1 µm in diameter and pores ranging from 3 to 10 µm (Fig. 2E,F). However, fibrin gels alone (without cells) were not dissolved in the culture medium. Even when aprotinin (serine-threonine protease inhibitor) was applied to the culture medium, scaffolds were degraded by only 60% on day 7 (Fig. 2G).

**Figure 3:**
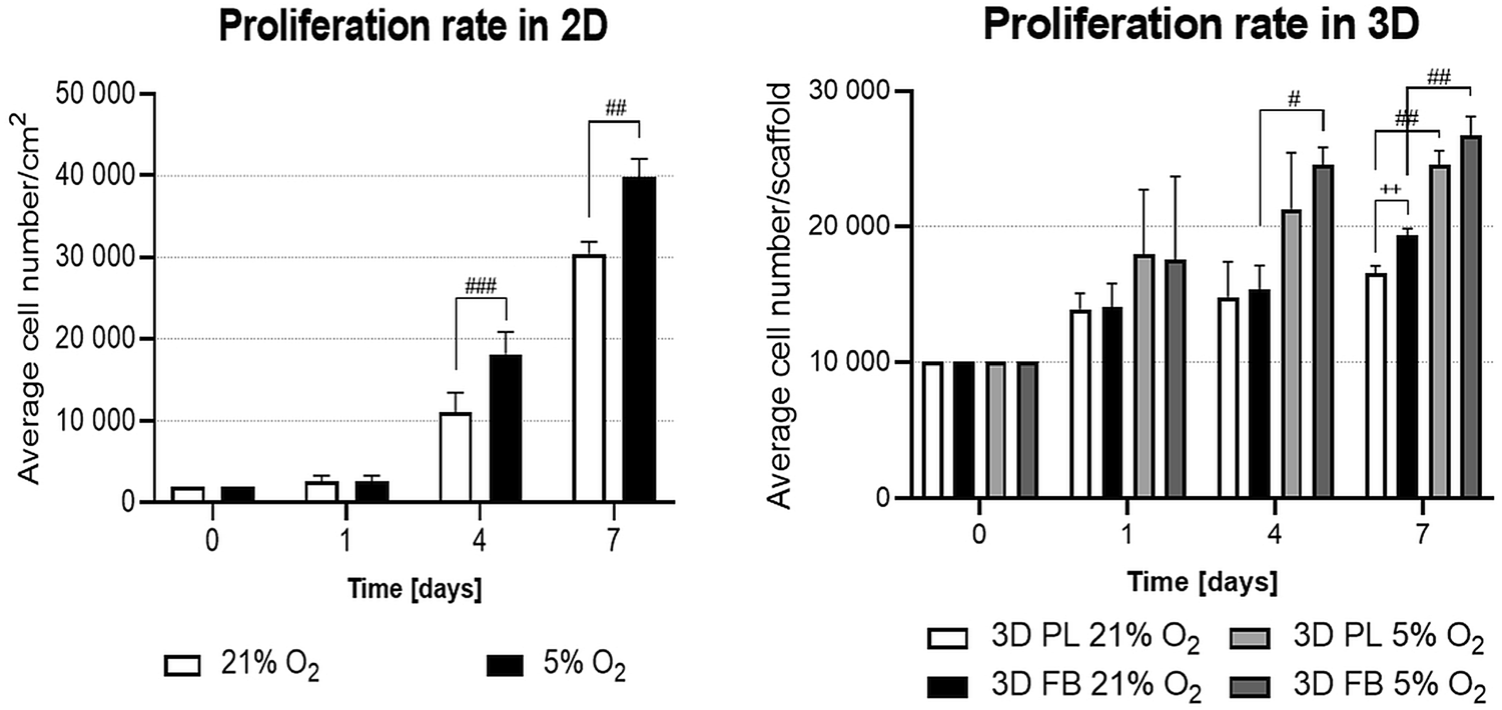
Analysis of WJ-MSCs proliferation rate in 2D and 3D hydrogel scaffolds cultured in different oxygen conditions. The results are presented as a mean ± SD. Statistical significance was determined using parametric Student’s test and non-parametric Mann–Whitney test for unconfirmed normality distribution (for *Proliferation rate in 2D*) and One Way ANOVA followed by Tukey’s multiple comparisons test (for *Proliferation rate in 3D*) (n = 18; #*p* < 0.05; ##*p* < 0.01; ###*p* < 0.001; +  + *p* < 0.01).

The WJ-MSCs' growth rate was analyzed in various spatial (2D and 3D) and aerobic (21% and 5% O_2_) conditions during the 7-day culture. Analysis showed a linear increase of proliferation in both types of cultures (2D and 3D) which occurred earlier in 5% O_2_ than 21% O_2_. Proliferation rate was significantly higher in 5% O_2_ in both 2D and 3D cell cultures but the statistically significant difference appeared much earlier in 2D. The scaffold type did not make a notable difference in the proliferation rate under 5% O_2_ (26 735 ± 1 388 cells per scaffold for FB vs. 24 477 ± 1 112 per scaffold for PL) but the final number of cells in FB scaffolds cultured at 21% O_2_ was slightly greater than in PL scaffolds (19 322 ± 506 per scaffold for FB vs. 16 536 ± 563 per scaffold for PL, Fig. 3).

**Figure 4:**
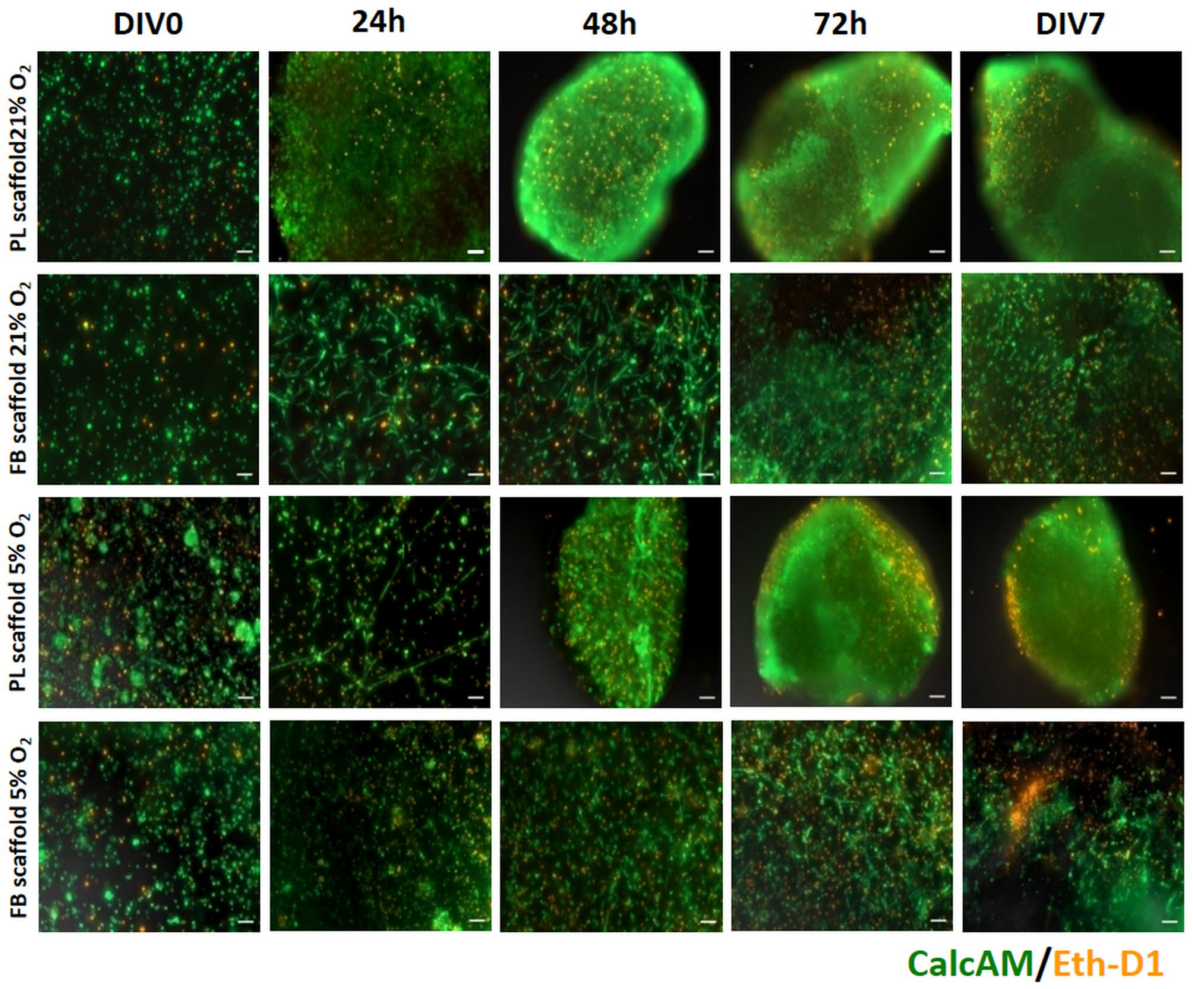
Cell viability in the 3D platelet lysate (PL) and fibrin (FB) scaffolds with WJ-MSCs cultured in 21% O_2_ or 5% O_2_ during 7 days (DIV) of culture. Living cells were stained with calcein AM (green), dead cells were stained with ethidium homodimer 1 (Eth-D1, orange). The areas of acquired images were the same for every investigated scaffolds. Scale bars = 100 µm.

Using fluorescent markers—calcein AM (green) and ethidium homodimer-1 (orange), the distribution of live and dead cells was determined during the 7-day culture in different scaffolds: FB or PL, each under two aerobic conditions. The cells encapsulated in both types of hydrogel were mostly live over the entire time of culture and grew protrusions that were observable as early as 24 and 48 h and remained visible during the next 7 days of analysis, which indicates the cells could connect through the network. The dimensions of PL-based scaffolds were visibly reduced at 48 h post-cell encapsulation, most likely due to the degradation of the delicate structure and the partial dissolution of its components in the medium. The structure of the FB scaffolds was more durable compared to that of the PL scaffolds and retained its size throughout 7 days of culture (Fig. 4).

**Figure 5:**
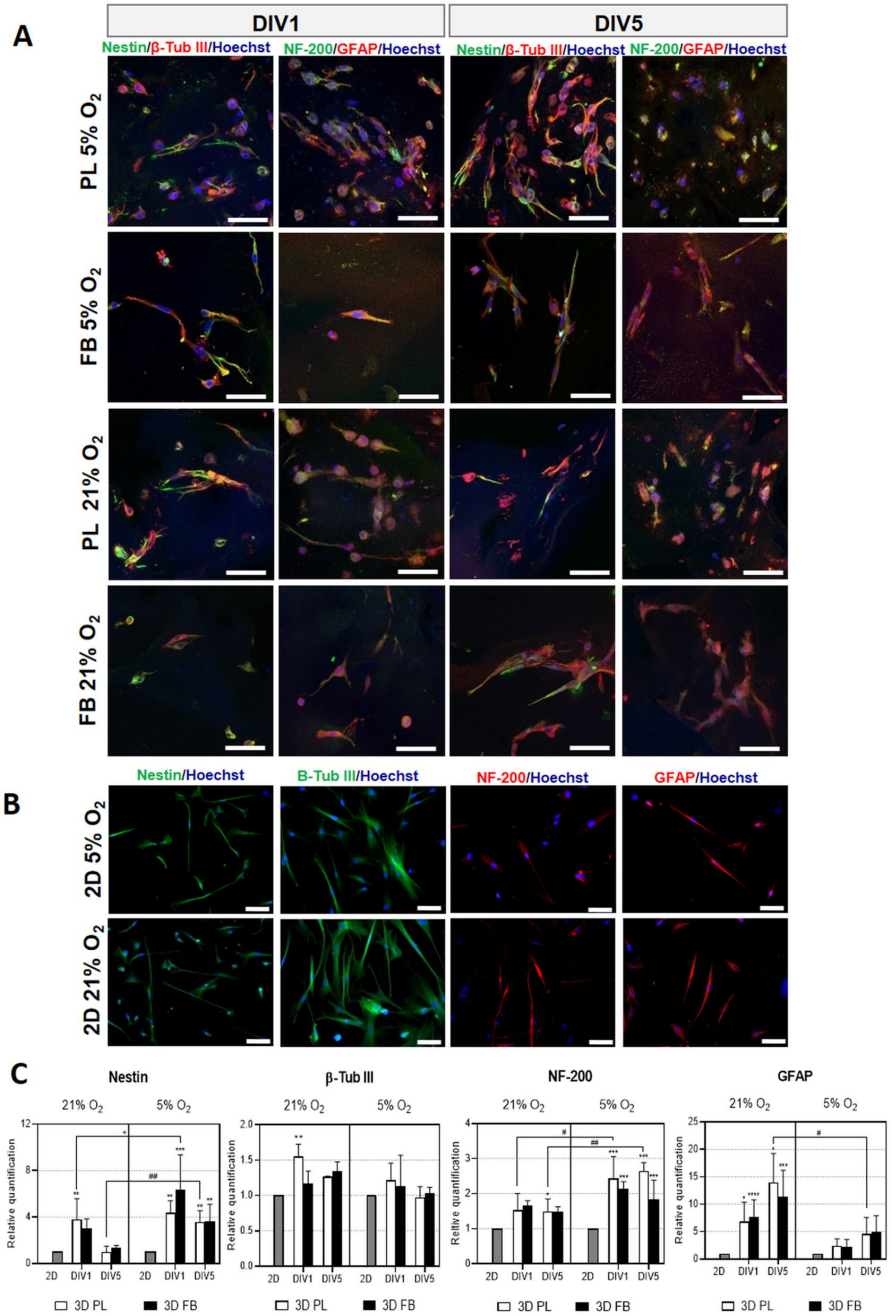
The expression of neural markers by WJ-MSCs preconditioned in the 3D platelet lysate-derived (3D PL) or fibrin (3D FB) scaffolds (**A**) or cultured on a standard plastic dish (2D) (**B**) after 1 or 5 days of culture in 21% O_2_ or 5% O_2_. Representative images of immunocytochemical analysis (**A**, **B**) and mRNA expression (**C**) of neural, neuronal and astrocytic markers (Nestin, β-Tubulin III, NF-200, GFAP, respectively). Scale bars = 100 µm. The relative quantification of mRNA expression was normalized to the reference gene ACTB. The results are presented in relation to the 2D control as a calibrator group (value 1, statistical significance marked by "*"). The results are presented as a mean ± SD. Statistical analysis was performed using One Way ANOVA followed by Tukey’s multiple comparisons test (n = 9; *p < 0.05; **p < 0.01; ***p < 0.001; ****p < 0.0001; #*p* < 0.05; ##*p* < 0.01; + *p* < 0.05), where "#" is a comparison 21% O_2_ and 5% O_2_ culture conditions while " + " is the statistical significance of mRNA expression level between PL and FB scaffolds.

Additionally, a comparison of 2D and 3D cultures for immunocytochemical analysis and expression of mRNA for neural markers showed that WJ-MSCs cultured in PL and FB scaffolds exhibited elevated expression of Nestin, β-Tubulin III, Neurofilament 200 (NF-200), and Glial Fibrillary Acidic Protein (GFAP) in both oxygen concentrations (21% and 5%). This effect was also observed after 5 days in vitro (Fig. 5A–C).

Expression profiles of selected growth factors and cytokines were determined for WJ-MSCs grown in in vitro 2D and 3D culture conditions. These profiles included 2D and 3D cultures under different oxygen-concentration conditions after 24 h and 5 days. The selected growth factors and cytokines were chosen for their strong association with survival, growth, differentiation of neural stem cells, and inflammatory response.

**Figure 6:**
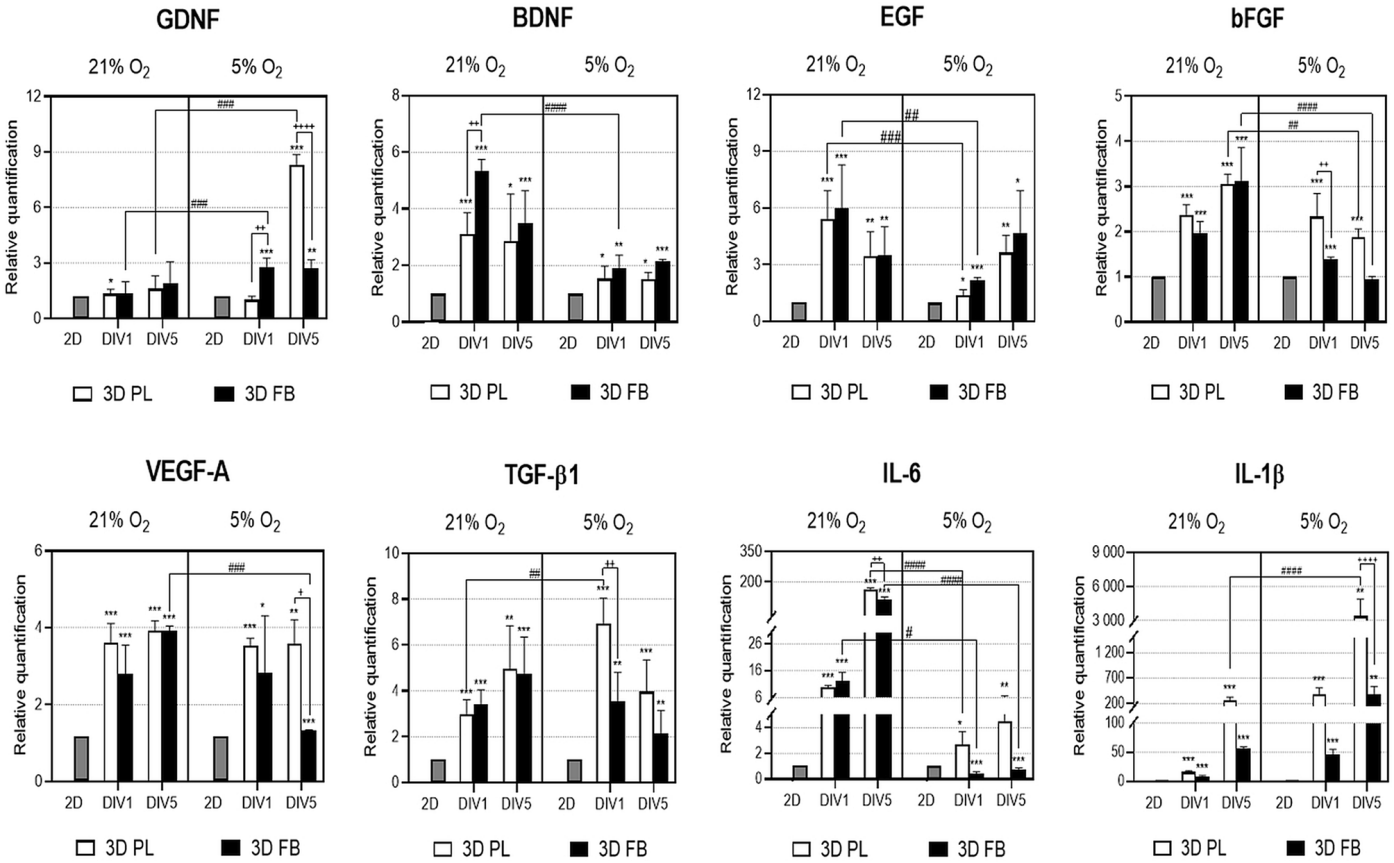
Analysis of mRNA expression of trophic factors and cytokines in WJ-MSCs encapsulated in 3D PL or 3D FB scaffolds in vitro (qRT-PCR). The relative quantification was normalized to the reference gene ACTB. The results are presented in relation to the 2D control as a calibrator group (value 1, statistical significance marked by "*"). The results are presented as a mean ± SD. Statistical analysis was performed using One Way ANOVA followed by Tukey’s multiple comparisons test (n = 9; *p < 0.05; **p < 0.01; ***p < 0.001; ##*p* < 0.01; ###*p* < 0.001; ####*p* < 0.0001; +  + *p* < 0.01; +  +  +  + *p* < 0.0001), where "#"is a comparison 21% O_2_ and 5% O_2_ culture conditions while " + "is the statistical significance of mRNA expression level between PL and FB scaffolds.

Comparative analysis showed significant changes in mRNA expression levels of the tested cytokines and growth factors after encapsulating the WJ-MSCs in the 3D scaffold structure. All the tested factors were up-regulated when cells where cultured in both FB and PL scaffolds. In all experimental groups, the applied 5% O_2_ culture condition resulted in elevated expression of Glial Cell-Derived Neurotrophic Factor (GDNF) and interleukin 1β (IL-1β), while atmospheric conditions were more favorable for Brain-Derived Neurotrophic Factor (BDNF) and interleukin 6 (IL-6) expression. Between DIV1 and DIV5 of culture in 5% O_2_, the mRNA levels for GDNF, BDNF, Epidermal Growth Factor (EGF), IL-1β, and IL-6 increased during culture time while mRNA for Vascular Endothelial Growth Factor A (VEGF-A), basic Fibroblast Growth Factor (bFGF), and Transforming Growth Factor beta 1 (TGF-β1) were down-regulated (Fig. 6).

### The assessment of neuroprotective effect of WJ-MSCs encapsulated in the scaffolds

**Figure 7:**
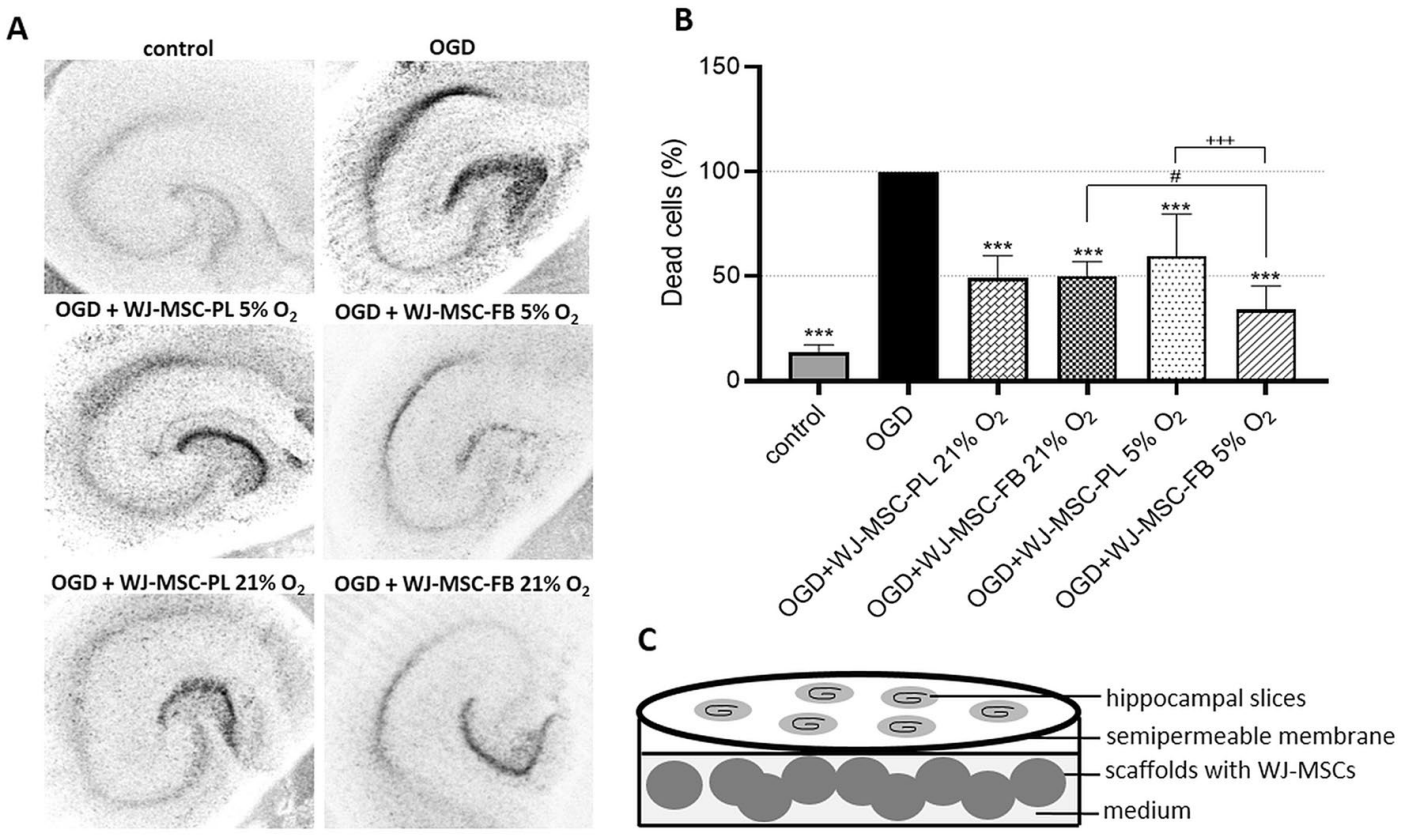
Assessment of the WJ-MSCs neuroprotective effect in the ex vivo experiments. The images present hippocampal slices in control conditions and after OGD procedure, dead cells stained with propidium iodide are black (**A**). The chart (**B**) represents the number of dead cells estimated from the sections of the hippocampus (in its damaged CA1 region), co-cultured with cells/scaffolds constructs (WJ-MSC-PL or WJ-MSC-FB) in 5% O_2_ or 21% O_2_. The results are presented as the mean ± SD (n = 12; *p < 0.05;***p < 0.001) compared with cell death after the OGD. Statistical analysis was performed using One Way ANOVA followed by Tukey’s multiple comparisons test. Statistically significant difference between 21% O_2_ and 5% O_2_ culture conditions is marked by "#" (#*p* < 0.05) and between PL and FB scaffolds is marked by " + " (+ +  + *p* < 0.001). The outline of indirect culture of organotypic hippocampal slices (control or after OGD) with WJ-MSCs encapsulated in the scaffolds is presented at (**C**).

A model of OGD in hippocampal organotypic slice cultures (OHCs) was used to evaluate ex vivo the neuroprotective properties of cell-hydrogel constructs (Fig. 7). Oxygen–glucose deprivation mimics an ischemic injury and the most sensitive cells in CA1 hippocampal region die immediately after the procedure. Co-culture with WJ-MSCs allows assessment of neuroprotective capacity by reducing the number of dead cells in this region. This experiment was designed to compare the degree of cell death and how it might be influenced by various types of WJ-MSCs co-cultures against tissue damage caused by OGD. Consequently, 100% of the cells in the CA1 region that were stained with propidium iodide were dead after OGD (Fig. 7A). This value was used to compare outcomes of various cultures. In all described variants where encapsulated WJ-MSCs were co-cultured with injured OHCs, a strong neuroprotective effect was exhibited (Fig. 7A,B). The strongest effect was observed after co-culture with WJ-MSCs seeded in FB under 5% O_2_ (OGD + WJ-MSC-FB 5% O_2_), with cell-death reduction as high as 34.6 ± 11.2%. In the remaining variants, WJ-MSCs reduced cell death by as much as 59.6 ± 19.9% (OGD + WJ-MSC-PL 5% O_2_), 49 ± 10.7% (OGD + WJ-MSC-PL 21% O_2_), and 50.4 ± 7.9% (OGD + WJ-MSC-FB 21% O_2_), respectively. In the control group (OHCs without OGD), spontaneous cell death due to culture in vitro as high as 13.6 ± 3.6% was observed (Fig. 7B).

**Figure 8:**
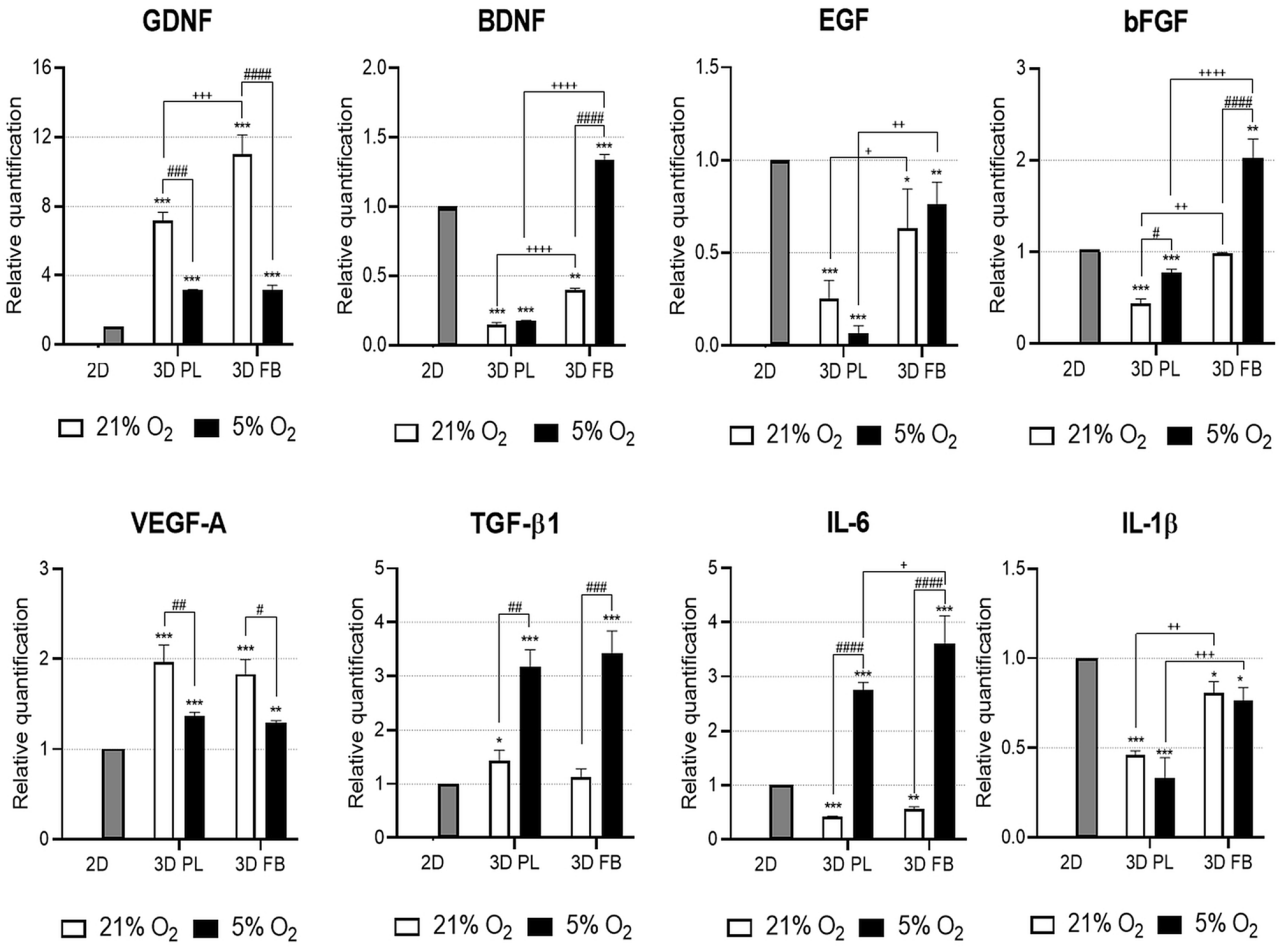
Analysis of mRNA expression of trophic factors and cytokines in WJ-MSCs encapsulated in 3D PL or 3D FB scaffolds after co-culture with OGD-damaged rat hippocampal slices (qRT-PCR). The relative quantification was normalized to the reference gene ACTB. The results are presented relative to the 2D control conditions as a calibrator group (value 1, statistical significance marked by "*"). The results are presented as a mean ± SD. Statistical analysis was performed using One Way ANOVA followed by Tukey’s multiple comparisons test (n = 9; *p < 0.05; **p < 0.01; ***p < 0.001; ****p < 0.0001; ##*p* < 0.01; ###*p* < 0.001; ####*p* < 0.0001; + *p* < 0.05; +  + *p* < 0.01; +  +  + *p* < 0.001; +  +  +  + *p* < 0.0001), where "#" is a comparison 21% O_2_ and 5% O_2_ culture conditions while " + " is the statistical significance of mRNA expression level between PL and FB scaffolds.

WJ-MSCs cultured in the scaffolds and then indirectly co-cultured with OHCs (Fig. 7C), when compared to parallel 2D cultures, revealed increased mRNA expression of GDNF, TGF-β1, and VEGF-A (Fig. 8). At the same time, WJ-MSCs in hydrogels exposed to the inflammatory environment of injured hippocampal slices significantly decreased expression of IL-1β while exhibiting increased expression of IL-6. This effect was potentiated by FB (but not PL) scaffolds and 5% O_2_ culture conditions (Fig. 8). However, statistically significant up-regulation of mRNA expression levels of BDNF and bFGF were observed only in FB scaffolds cultured in physiological normoxia (5% O_2_).

## Discussion

The current need to establish precise protocols for culture, isolation, phenotypic verification, and biosafety for acquired stem cells exists and includes the need for protocols governing enhancement of therapeutic properties and survival after transplantation^5^. Stem cells are exposed to a strong recipient immune response after transplantation; this immune response can significantly hinder survival and regeneration of damaged tissue. Specific carriers or scaffolds that protect transplanted cells against attack from the recipient immune system and enhance the regenerative properties of stem cells are being developed to overcome this situation.

In this study, PL- and FB-based scaffold systems were used to provide support for cells, sustain their migratory capabilities, and enhance their pro-regenerative profile. PL and FB scaffolds have been used previously for skeletal muscle regeneration and to develop clinical strategies for bone repair^23,24^ but not with WJ-MSCs for neuro-repair studies. Here, we have used such bio-scaffolds to estimate neuroprotective and anti-inflammatory properties of WJ-MSCs and to compare the utility of different scaffolds for possible transplantation to the injured Central Nervous System (CNS). The PL-based scaffolds used in this study were made from the solution of the platelet lysate, which contains less fibrinogen than FB scaffolds (approximately 0.43 mg/mL in PL, as provided by manufacturer^22^ vs. 5 mg/mL of fibrinogen in fibrin scaffolds). Therefore, the fibrin fibers in PL scaffolds are thinner, the entire structure is softer, and more susceptible to cellular remodeling (Fig. 2A,B). Also, these scaffolds made from 5 mg/mL fibrinogen and 2 U/mL of thrombin, giving them elasticity that closely resembles that of brain tissue (below 1 kPa)^25^. Cells originally attached to the scaffolds are released eventually in a remodeling process triggered by degradation of fibrin fibers (Fig. 2G). The remodeling capability of the scaffold biomaterials by encapsulated cells is an important factor concerning migration of the cells from scaffold to injured tissue; this same capacity for remodeling influences efficacy of neural differentiation^26^.

However, to maintain as close a match as possible to native-like conditions in vitro microenvironment, would require appropriate oxygen tension, typical for neural stem cell niche as well as mentioned above 3D culture. Several earlier reports document variances of oxygen tension in the neural stem cell niche between 1 and 8%^27,28^, thus for this study, in vitro physiological normoxia conditions of 5% O_2_were established. It has been reported, that cell culturing in 21% O_2_ (still routinely used for the majority of stem cell cultures) represents non-physiological conditions^4,29^. We have previously demonstrated that low oxygen level during culture (5% O_2_) is optimal for the maintenance of these cells and influences their therapeutic competence by enhancing expression of neuroprotective and anti-inflammatory factors. Moreover, in our previous studies, we showed that the physiological normoxia of 5% O_2_ did not cause adverse karyotypic changes in MSCs in contrast to long-lasting culture in atmospheric oxygen conditions^4^, which was shown to inhibit growth and differentiation of marrow-derived MSCs^30^. Culturing in lower oxygen concentrations produces a positive effect on the phenotypic characteristics of cells. A number of studies show that the use of 3–10% O_2_ stimulates the proliferative potential of mesenchymal stem cells and inhibits their senescence processes during in vitro culture^4,31^. Here, we have confirmed that, for 2D standard cultures, physiological normoxia significantly improved proliferation and this was potentiated further in both types of scaffolds (Fig. 3). For these experiments, we used a specific hypoxia/normoxia chamber (*Xvivo System*, Biospherix) with an adjustable O_2_ level that provides a constant oxygen concentration during cell incubation and manipulation, including passaging and medium exchange.

A mesenchymal stem cell 3D encapsulation technology was shown to be important for providing biomimetic microenvironment for the cells^32^. During our on-going 7-day culture, cell spreading and viability were sustained (Fig. 4). However, in the case of PL hydrogel, we observed that the dimensions of the scaffold had already begun to decrease after only 48 h of culture (Fig. 4). The most likely because of this shrinkage is believed to be the pulling forces MSCs generate; shrinkage is less problematic in FB scaffolds due to their higher concentration of fibrinogen^33^. Fluorescent images suggest that WJ-MSCs, while remodeling the scaffold, were migrating across the 3D hydrogel, however support from the scaffold remained effective. Cell mortality was low and observed mainly at the edges of the scaffolds. Such observations suggest both the PL- and FB-hydrogel scaffolds used in this study provide optimal supporting conditions for biomimetic cell culture.

When comparing 2D and 3D cell culture conditions, it was observed that in 3D both scaffold types (PL and FB) enhanced neuronal and glial differentiation, as revealed by Nestin, β-Tubulin III, NF-200, and GFAP mRNA and protein expression (Fig. 5A–C). This suggest that biomaterials of human origin provide suitable biomimetic surroundings sustaining WJ-MSCs' neural differentiation. It was also observed that it is possible to alter the direction of differentiation of WJ-MSCs by manipulating oxygen levels. With oxygen concentration at 21% glial differentiation (GFAP expression) was promoted. Physiological normoxia promoted the neural stem cell marker, Nestin, and NF-200 (marker for more differentiated neurons) expression. Francis and colleagues^34^ have shown that hypoxic preconditioning increases the neurogenic capability of neural progenitors derived from human embryonic stem cells (hESCs). Furthermore, MSCs isolated from rat bone marrow exhibit increased expression of GFAP in 21% O_2_ while lowered oxygen tension caused increased expression of Nestin and Neuron-specific class III beta-tubulin (TUJ-1)^35^.

It was reported, that the stiffness of the growth substrate can influence differentiation of induced Pluripotent Stem Cells (iPSCs), where without additional neurogenic factors compliant, soft hydrogel promoted expression of neuronal markers in neural progenitors derived from iPSC^36^. Fibrin hydrogel culture provides softer culture conditions (elasticity of 3 mg/mL fibrin hydrogel is around 3 kPa); in comparison, the elasticity of standard polystyrene (plastic dish material) is about 10^5^–10^6^ kPa^37^. The expression of neural markers slightly decreases at day 5 of culture, possibly due to aggregation of cells growing inside the hydrogel, especially in PL scaffolds, where the stiffness of the hybrid bio-construct may increase, therefore may promote non-differentiation state. Three-dimensional constructs are determined by the targeted tissue and transplantation method. Scaffolds should be adjusted to their future tissue-specific application with respect to composition, shape, biodegradability, and flexibility of the biomaterial; doing so will likely create biomimetic conditions most similar to the endogenous niche of the stem cells^32,38–40^.

This thesis is supported by recent findings of an in vitro and in vivo experimental model of limb ischemia. In that study, hydrogel composed of pooled PL was found to stimulate pro-angiogenic activity by promoting human MSCs growth and invasion in a 3D environment. It also enhanced endothelial cell sprouting alone and in co-culture with MSCs^41^. However, other research found that human PL did not improve cell survival within hydrogel constructs beyond 2 weeks post-implantation^42^. This finding may be explained as being dose dependent as shown in an experiment with MSCs derived from dental pulp. In this case, the appropriate concentration of PL enhanced proliferation of dental pulp stem cells in vitro and produced a significantly positive regenerative effect in vivo^43^. Review of available literature supports the idea of using PL as a non-zoonotic adjuvant for cell culture in clinical research.

It was proved that regeneration of bone and cartilage after application of MSCs combined with biomaterials takes place due to tissue replacement^44–46^. In case of neurological disorders, replacement mechanism of regeneration is disputable, however neuro-therapeutic potential of MSCs applied within hydrogel was observed due to their secretory properties, such as production of immunomodulatory or neurogenic factors^47^.

We have shown that WJ-MSCs preconditioned by physiological normoxia (5% O_2_) and cultured in 3D hydrogel scaffolds, as compared to WJ-MSCs cultured in 21% O_2_ and 2D conditions, show increased neuroprotective effect, can modulate the inflammatory response, and enhance the regenerative process (Figs. 6, 7, 8). In our study organotypic hippocampal slices, temporally deprived of oxygen and glucose (OGD) reveal high neuronal death in CA1 region, but when co-cultured with WJ-MSCs encapsulated in bio-scaffolds showed a statistically significant reduction in cell mortality in this hippocampal region. This neuroprotective effect was obtained in the culture conditions without any direct contact between the hippocampal slices and the WJ-MSCs, thus strongly suggesting paracrine mechanism of action.

Such effect was previously confirmed by the research of Sarnowska et al.^48^, in which increased cell survival in the hippocampal CA1 region was observed following OHCs and Human Umbilical Cord Blood-derived Neural Stem/Progenitor Cells (HUCB-NSCs) co-culture. Our previous studies have shown that WJ-MSCs cultured in 2D showed a significantly reduced neuronal mortality by as much as 25%^10^. In the described study, results are similar. The co-culture of injured OHCs with encapsulated WJ-MSCs resulted in neuroprotection. The neuroprotective effect was most evident when cells were preconditioned under 5% O_2_ and encapsulated in fibrin scaffolds (34.6 ± 11.2%). The neuroprotective properties of WJ-MSCs cultured in atmospheric conditions were similar in both applied types of scaffolds.

Recently, our group demonstrated that human Wharton's Jelly-derived mesenchymal stem cells could secrete several anti-inflammatory, anti-apoptotic, angiogenic, and neurogenic factors^4,10^. Moreover, it has been reported that MSCs have the ability to change the phenotype on immunomodulatory (MS2) or proinflammatory (MS1) factors by stimulation of corresponding Toll-like receptors^49^. Here, we proved that cells preconditioned in a 3D environment sustain these neuroprotective characteristics.

Assessment of the expression profiles of several neurotrophins, growth factors, and cytokines such as GDNF, BDNF, EGF, bFGF, VEGF-A, TGF-β1, IL-6, and IL-1β in WJ-MSCs suspended in hydrogels revealed that, in the 3D microenvironment, the expression level of mRNA for all of these genes was up-regulated. This effect was observed after only 24 h of 3D culture and it lasted for several days in vitro (Fig. 6). The observed results were also confirmed in ex vivo studies with organotypic cultures, verifying the response of WJ-MSCs encapsulated in hydrogel scaffolds to the injured nervous tissue environment. After 24 h of co-culture WJ-MSCs with OGD-treated rat hippocampal slices, mRNA expression levels of several neurotrophins responsible for neuroprotective properties (GDNF and VEGF-A, for example) were statistically higher than that observed in WJ-MSCs cultured in 2D. That was not the case for the growth factors such as BDNF, EGF, and bFGF in the presence of injured tissue; their expression was down-regulated more in the 3D environment than in the 2D control conditions. However, preconditioning cells in fibrin scaffolds and in 5% O_2_ rescued this effect. Additionally, the in vitro cultures of WJ-MSCs encapsulated in PL and FB scaffolds exhibited enhanced expression of pro-inflammatory IL-6 and IL-1β, but expression upon exposure to injured nervous tissue produced different results. When so exposed, these WJ-MSCs in bio-hydrogels showed decreased mRNA expression of pro-inflammatory IL-1β and elevated expression of anti-inflammatory TGF-β1. These expression variations were evident to a greater degree in 3D than in 2D conditions (Fig. 8). These results suggest a potential vasculoprotective effect, reduction of the inflammatory response exerted by WJ-MSCs-cultured scaffolds as a result of stimulation by the damaged tissue environment, and strong influence of cells' preconditioning parameters.

We have seen that 3D hydrogel constructs are more effective in promoting mRNA expression and protein production of soluble immune-related factors than standard 2D conditions^44^. In MSCs cultured in 3D scaffolds, enhanced expression of anti-inflammatory cytokines (Prostaglandin E2—PGE-2, Tumor necrosis factor-inducible gene 6—TSG-6) and reduced expression of pro-inflammatory cytokines (IL-6, Monocyte Chemoattractant Protein 1—MCP-1) was observed^50^. Moreover, WJ-MSCs encapsulated in 3D scaffolds might also reduce the immune response to subcutaneous implantation ^44^.

It is interesting to note that, in our study, the level of pro-inflammatory cytokine IL-6 in cells cultured under normoxic (5% O_2_) conditions is much less elevated during 3D culture than in bio-scaffolds growing in atmospheric conditions (21% O_2_). However, this effect is completely opposite when WJ-MSCs embedded in hydrogels are exposed to damaged neural tissue, suggesting stimulation of immunomodulatory properties by combined features of cellular preconditioning together with factors secreted by the microenvironment of injured tissue.

## Conclusions

Cells’ preconditioning before transplantation is necessary to adapt the molecular sensing of oxygen levels by the cells to provide specific conditions closest to those encountered in their natural niche. Both scaffold types used in this study enriched the regenerative properties of WJ-MSCs. Thus, the most beneficial approach appears to be maintenance of cells in culture under physiological normoxia and their subsequent transplantation into an environment that will be protective for the cells, such as 3D human-protein-derived hydrogel scaffolds with mechanical properties similar to brain nervous tissue. More evaluation is still needed to affects cell viability after transplantation and the cell/scaffold hybrids could help to decrease the cells' mortality. We claim that the final therapeutic properties of transplanted cells depend on the combined effects of the cells' preconditioning and factors secreted by the microenvironment of injured tissue.

## Methods

All experiments were carried out in accordance with relevant guidelines and regulations.

### Cells isolation and culture conditions

Human umbilical cords were acquired from full-term pregnancies, after obtaining informed consents of all mothers and in accordance with approval by the Bioethics Commission of the Medical University of Warsaw (KB/213/2016).

Cords were briefly immersed in sterile Phosphate-Buffered Saline (PBS, Gibco) supplemented with penicillin–streptomycin (1:100, Gibco) then cut into 2–3 mm slices using a sterile scalpel. Using a biopsy punch (Miltex, GmbH) and avoiding blood vessels, cylindrical fragments 3 mm in diameter were cut from the slices. Wharton’s Jelly fragments were placed into 6-well culture plates with 1 mL of growth medium in each well. This growth medium consisted of Dulbecco's Modified Eagle Medium (DMEM, Macopharma) supplemented with 10% human platelet lysate (Macopharma), 1 mg/mL of glucose (Sigma-Aldrich), 2 U/mL heparin (Sigma-Aldrich), and Antibiotic–Antimycotic Solution (AAS*,* 1:100, Gibco). Cells that migrated out of the fragments were cultured up to 70% confluence in a humidified incubator at 21% O_2_, 5% CO_2_, and 37 °C or in a closed system that maintains constant oxygen concentration of 5% O_2,_ 5% CO_2_ at 37 °C *(Xvivo System,* BioSpherix)*.*

The growth medium was replaced every 2 days. When the cells reached a suitable confluence, the cultures were passaged using Accutase Cell Detachment Solution (Becton Dickinson).

### Scaffolds preparation

Two different types of scaffolds were prepared. One was made from human platelet lysate (PL, Macopharma) and the second from human plasma fibrinogen (5 mg/mL, Sigma-Aldrich) here after referred as PL and FB, respectively. Active coagulation factor II (thrombin) was used as a cross-linker to prepare the scaffolds. Thrombin is a glycoprotein (α2-globulin) with proteolytic properties involved in the transformation of soluble fibrinogen into insoluble fibrin. Thrombin (Sigma-Aldrich) was diluted in Tris-Buffered Saline (TBS, Sigma-Aldrich) and supplemented with calcium chloride (CaCl_2_) to the final concentrations of 2 U/mL of thrombin and 250 µM CaCl_2_. To encapsulate the cells in the scaffolds, WJ-MSC were re-suspended in 50 µL of platelet lysate or fibrinogen solution in the presence of the fibrinogen degradation inhibitor, aprotinin (10 µg/mL, Sigma-Aldrich). Each scaffold contained 10 000 cells. Next, 50 µL of thrombin solution was transferred to Petri dishes as a drop and mixed with 50 µL of cells suspended in platelet lysate or fibrinogen. Fresh cell/scaffold constructs were incubated for 1 h at 37 °C at 5% CO_2_, 21% O_2,_ or 5% O_2_ (*Xvivo System,* BioSpherix). Next, the scaffolds were transferred into the 6-well plates containing 2 mL of growth medium in each well. The growth medium consisted of DMEM (Macopharma) supplemented with 10% human platelet lysate (Macopharma), 1 mg/mL glucose (Sigma-Aldrich), 2 U/mL heparin (Sigma-Aldrich), and AAS (1:100, Gibco) with addition of 10 µg/mL aprotinin. The cell/scaffold hydrogels were cultured as floating cultures.

### Fibrin network formation analysis

The internal structure of scaffolds was labeled by adding a portion (1:100) of fibrinogen from human plasma conjugated with Alexa Fluor 546 (Molecular Probes) to a freshly prepared fraction of fibrinogen or platelet lysate. Empty scaffolds were made as follows: thrombin (50 µL) was transferred on the microscopic slide, mixed with 50 µL of labeled PL or fibrinogen, cover slide closed, and incubated in 37 °C for 1 h. Then, network formation was visualizedin the confocal LSM510 system (Zeiss). Measurements of fiber diameter and pore size wereperformed using Zen software (Zeiss).

### Scaffold degradation

Alexa Fluor 546-labeled fibrinogen (1:100) was used to prepare empty FB scaffolds or scaffolds with cells for incubation in 96-well plates with culture medium. At days 1, 3, and 7 of culture, the culture medium was collected and fluorescence measured using Omega Plate Reader (BMG LABTECH). The intensity of fluorescence of culture medium containing degraded labeled fibrinogen was calculated in the relation to the fluorescence of complete dissolved scaffold (100%) that was made using trypsin.

### WJ-MSCs migration

WJ-MSCs were labeled by adding 10 mM CMFDA—5-chloromethylfluorescein diacetate (ThermoFisher Scientific) into the culture flasks and incubated for 1 h at 37 °C. After this time cells were detached, centrifuged, and suspended either with PL or fibrinogen solution. Cell/scaffold constructs were made as follows: a drop of 50 µL of thrombin was placed on the bottom of each well in 6-well plates then mixed with cells re-suspended in 50 µL of PL or fibrinogen solution in the presence of aprotinin (10 µg/mL). After 1 h of incubation at 37 °C and 5% CO_2_, 21% O_2,_or 5% O_2_, fresh medium was added carefully to each well with one scaffold attached to the bottom of the dish. WJ-MSCs migration out of the scaffold structure was analyzed after 7 days using contrast phase and fluorescent microscope with AxioCam MRc5 (Zeiss) digital camera and Zen 2012 software (Zeiss).

### Live/dead assay

At 5th passage, WJ-MSCs cultured in hydrogel scaffolds were analyzed by LIVE/DEAD Viability/Cytotoxicity Kit (Invitrogen). For qualitative analysis, calceinacetoxymethyl (calceinAM) for live cells and ethidium homodimer (EthD-1) for dead cells were added in 1:1000 concentrations in PBS. Scaffolds were transferred into 35/10-mm glass-bottom dishes and incubated for 20 min, protected from light. Then, the PBS was replaced by fresh growth medium, labeled cells were immediately observed with Cell Observer SD System with Axio Observer Z.1 microscope (Zeiss) and images of whole scaffolds were acquired. Scaffolds with WJ-MSCs cultured under 21% O_2_ and 5% O_2_were analyzed 1 h after preparation and again at the 24-, 48-, and 72-h marks, with final analysis on day 7 of culture.

### Proliferation analysis

At 3rd-5th passage of WJ-MSCs, cells were encapsulated in the scaffold structure as described above and treated by enzymatic digestion of collagenase NB 4 Standard Grade solution (Serva) at a final concentration of 20 U/mL prepared in PBS. After 30 min of incubation under 5% CO_2,_ 21% O_2,_ or 5% O_2_ at 37 °C, the cells were washed twice in PBS and centrifuged for 5 min at 1500 rpm. Next, the supernatant was removed and the pellets frozen in − 80 °C for further analysis.

The proliferation rate was analyzed 1 h after preparation of cell/scaffold constructs, after 24 h, and on 4 and 7 days of culture in both 3D and 2D by using the CyQuant Cell Proliferation Assay Kit (Invitrogen) according to manufacturer's protocol. The cell pellets were thawed at room temperature and suspended in 200 µL of prepared cell-lysis buffer containing green fluorescent dye which bonds to cellular nucleic acids. After 5 min of incubation in the dark, samples were transferred to 96-well microplates and fluorescence was measured at 480 nm using Omega Plate Reader (BMG LABTECH). The curve of cell growth in each type of scaffold and in 2D conditions was plotted over time at X-axis and cell countat Y-axis. All experiments were performed for three independent isolations.

### Immunocytochemistry (ICC)

For ICC, WJ-MSCs in PL and FB scaffolds were fixed with 4% paraformaldehyde (PFA) for 20 min at room temperature after days 1 and 5 of culture as well as WJ-MSCs cultured in 2D. After fixation, scaffolds were rinsed with PBS and submerged in 30% saccharose for dehydration. In the next step, scaffolds were submerged in a compound of 7.5% gelatin and 10% saccharose, kept on dry ice for polymerization, and then stored at − 80 °C. Frozen scaffolds were cut into 20-µm slices using a cryostat and deposited on silanized glass slides. To eliminate non-specific binding and facilitate membrane permeabilization, the samples (2D and 3D) were incubated for 1 h at room temperature with 10% goat serum containing 0.25% triton X100. After washing with PBS, scaffolds and 2D cells were incubated with primary antibodies (listed in Supplementary Table 1) in the permeabilization/blocking solution overnight at 4 °C. The following day, after washing with PBS, cells were incubated with appropriate fluorochrome-conjugated secondary antibodies for 1 h at room temperature followed by three washes with PBS. Cell nuclei were stained with 5 μM Hoechst 33258 for 20 min at room temperature. After rinsing with PBS, Fluorescent Mounting Medium (Dako) was added to the scaffolds and 2D cells, which were then covered with glass coverslips. Images were made using the confocal LSM780 system (Zeiss) and Axio Vert.A1 (Zeiss) fluorescent microscope with AxioCam MRc5 (Zeiss) digital camera and Zen 2012 software (Zeiss).

### Organotypic hippocampal culture (OHC)

Organotypic hippocampal slice cultures were obtained as described previously^10^. In summary, the hippocampi were obtained from 7-day-old Wistar rats after decapitation and were cut into 400-µm slices using a McIlwain tissue chopper immediately after isolation. Hippocampal slices were washed in PBS, transferred to semipermeable membranes (Millicell CM, Millipore), and placed in 6-well plates. In each well, 0.9 mL of culture medium was inserted. This medium consisted of DMEM (Gibco), horse serum (25%; Sigma-Aldrich), HEPES-Buffered Hanks Balanced Salt Solution (HHBSS, 25%; Gibco), 1 M HEPES (Gibco), 5 mg/mL glucose (Sigma-Aldrich), and antibiotics (1% amphotericin B and 0.4% penicillin–streptomycin) (Gibco) at 35 °C. During the following 5 days of culture, the serum concentration was gradually lowered until a serum-free medium containing DMEM (Gibco), HBSS, 1 M HEPES, glucose, and antibacterial–antimycotic solution was achieved. On the 5th day in vitro, hippocampal slices were exposed to Oxygen–Glucose Deprivation (OGD) conditions and used for further co-culture experiments.

### Oxygen–glucose deprivation procedure

For the OGD procedure, hippocampal slices were placed in deoxygenated Ringer solution (Sigma-Aldrich) supplemented with 10 mM mannitol (Sigma-Aldrich). Membranes with OHCs were immediately transferred to an anaerobic chamber and saturated with 95% N_2_ and 5% CO_2_. The slices were kept at 35 °C in an oxygen-free atmosphere for 40 min in order to mimic an ischemic injury. The control OHCs were maintained in standard conditions. Cell death in the CA1 hippocampal region was analyzed 24 h after co-culture with WJ-MSCs to verify the neuroprotective effect of MSCs.

### Co-culture of WJ-MSCs in 2D and 3D with OHC slices

Immediately after the OGD procedure, the membranes with the hippocampal slices were transferred to 6-well plates with WJ-MSCs cultured as 2D or 3D hydrogel scaffolds. During the 24 h of co-culture in serum-free medium (as mentioned previously), OHCs and WJ-MSCs were cultivated without direct contact with the cells placed under the semipermeable membranes containing hippocampal slices.

The next day, scaffolds were treated by enzymatic digestion of collagenase solution as mentioned previously and pellets and 2D cultures were collected for mRNA analysis. To determinate cell death in the CA1 hippocampal region, the fluorescent marker, propidium iodide (PI; Sigma-Aldrich), was used. This marker (1.4 µg/mL) was added to the culture medium for 1 h and then labeled cells in OHCs were immediately imaged using a confocal microscope (Carl Zeiss LSM 510) with ZEN software.

### Quantitative reverse transcription polymerase chain reaction (qRT-PCR) analysis

Expression of mRNA was calculated and analyzed from WJ-MSCs samples derived from three independent donors and performed in three replicates. Fenozol Reagent (A&A Biotechnology) was used to isolate mRNA from cells cultured in 2D and 3D conditions. Each sample of prepared material was treated with 1 U/mL DNase (Clean-Up RNA Concentration, A&A Biotechnology) and mRNA purity was evaluated by reading absorbance in a NanoDrop ND-1000 spectrophotometer. The High Capacity RNA-to-cDNA Kit (Applied Biosystems) was used for the preparation of cDNA, according to the manufacturer’s protocol. Quantitative RT-PCR (qRT-PCR) was performed on the cDNA samples using the 7500 Real-Time PCR System (Applied Biosystems), SYBR Green PCR Master Mix (Life Technologies), and gene-specific primers listed in Supplementary Table 2. Expression level of Actin Beta (ACTB) was used as an internal control and the final expression was calculated using the 2^−ΔΔCt^ method. The results were expressed as the normalized fold expression for each gene and values are presented relative to the 2D control conditions as a calibrator group for each aerobic culture condition.

### Microscope imaging

The visualizations were performed in the Laboratory of Advanced Microscopy Techniques, Mossakowski Medical Research Centre, Polish Academy of Sciences.

### Statistics

Statistical evaluation was based on a minimum of 3–5 independent experiments. Statistical analysis was performed using GraphPad Prism 8 software. Mean ± standard deviation (SD) was calculated for all samples and statistical significance was determined using the parametric Student’s t-test and non-parametric Mann–Whitney test for unconfirmed normality distribution, or analysis of variance (ANOVA) followed by the Tukey’s multiple comparisons test. A value of *p* < 0.05 was considered significant.

## Supplementary information

Supplementary file1

## References

[1] Stoltz JF (2015). Stem cells and regenerative medicine: myth or reality of the 21th century. Stem Cells Int..

[2] Baraniak PR, McDevit TC (2010). Stem cell paracrine actions and tissue regeneration. Regen. Med..

[3] Herberts CA, Kwa MSG, Hermsen HPH (2011). Risk factors in the development of stem cell therapy. J. Transl. Med..

[4] Lech W (2016). Phenotypic, functional, and safety control at preimplantation phase of MSC-based therapy. Stem Cells Int..

[5] Mallis P, Stavropoulos-Giokas C, Michalopoulos E (2019). Introduction to the special issue on stem cell and biologic scaffold engineering. Bioengineering.

[6] Chen F, Liu X (2016). Advancing biomaterials of human origin for tissue engineering. Prog. Polym. Sci..

[7] Chatzistamatiou TK (2014). Optimizing isolation culture and freezing methods to preserve Wharton's jelly's mesenchymal stem cell (MSC) properties: an MSC banking protocol validation for the Hellenic Cord Blood Bank. Transfusion.

[8] Christodoulou I, Kolisis FN, Papaevangeliou D, Zoumpourlis V (2013). Comparative evaluation of human mesenchymal stem cells of fetal (Wharton's Jelly) and adult (adipose tissue) origin during prolonged in vitro expansion: considerations for cytotherapy. Stem Cells Int..

[9] Drela K (2016). Enhanced neuro-therapeutic potential of Wharton’s Jelly-derived mesenchymal stem cells in comparison with bone marrow mesenchymal stem cells culture. Cytotherapy.

[10] Obtulowicz P, Lech W, Strojek L, Sarnowska A, Domanska-Janik K (2016). Induction of endothelial phenotype from Wharton Jelly-derived MSC and comparison of their vaso- and neuro-protective potential with primary WJ-MSC in CA1 hippocampal region ex vivo. Cell Transplant..

[11] Yuan H (2019). Introducing the language of “relativity” for new scaffold categorization. Bioengineering.

[12] O'Brien FJ (2011). Biomaterials & scaffolds for tissue engineering. Mater. Today.

[13] Salgado AJ (2013). Tissue engineering and regenerative medicine: past, present, and future. Int. Rev. Neurobiol..

[14] Anitua E, Prado R, Orive G (2013). Endogenous morphogens and fibrin bioscaffolds for stem cell therapeutics. Trends Biotechnol..

[15] Ahmed TA, Dare EV, Hincke M (2008). Fibrin: a versatile scaffold for tissue engineering applications. Tissue Eng. Part B Rev..

[16] Lu P, Graham L, Wang Y, Wu D, Tuszynski M (2014). Promotion of survival and differentiation of neural stem cells with fibrin and growth factor cocktails after severe spinal cord injury. J. Vis. Exp..

[17] Robinson M, Douglas S, Willerth SM (2017). Mechanically stable fibrin scafolds promote viability and induce neurite outgrowth in neural aggregates derived from human induced pluripotent stem cells. Sci. Rep..

[18] Shichinohe H (2011). Biological features of human bone marrow stromal cells (hBMSC) cultured with animal protein-free medium-safety and efficacy of clinical use for neurotransplantation. Transl. Stroke Res..

[19] Pérez-Ilzarbe M (2009). Comparison of ex vivo expansion culture conditions of mesenchymal stem cells for human cell therapy. Transfusion.

[20] Doucet C (2005). Platelet lysates promote mesenchymal stem cell expansion: a safety substitute for animal serum in cell-based therapy applications. J. Cell Physiol..

[21] Sandri G (2015). Platelet lysate embedded scaffolds for skin regeneration. Expert Opin. Drug Deliv..

[22] Viau, S. *et al*. A Standardized and Characterized GMP-Compliant Human Platelet Lysate for Efficient Expansion of Human Bone Marrow Mesenchymal Stem Cells. 22nd Annual ISCT Meeting **18(6)**, SUPPLEMENT, S131. (poster) https://doi.org/10.1016/j.jcyt.2016.03.254 (2016)

[23] Santos SCNDS, Sigurjonsson ÓE, Custódio CA, Mano JFCDL (2018). Blood plasma derivatives for tissue engineering and regenerative medicine therapies. Tissue Eng. Part B Rev..

[24] Altaie A, Owston H, Jones E (2016). Use of platelet lysate for bone regeneration—Are we ready for clinical translation?. World J. Stem Cells.

[25] Duong H, Wu B, Tawil B (2009). Modulation of 3D fibrin matrix stiffness by intrinsic fibrinogen-thrombin compositions and by extrinsic cellular activity. Tissue Eng. Part A.

[26] Madl CM, LeSavage BL, Dewi RE, Lampe KJ, Heilshorn SC (2019). Matrix remodeling enhances the differentiation capacity of neural progenitor cells in 3D hydrogels. Adv. Sci. (Weinh)..

[27] Mohyeldin A, Garzón-Muvdi T, Quiñones-Hinojosa A (2010). Oxygen in stem cell biology: a critical component of the stem cell niche. Cell Stem Cell.

[28] Huang G (2017). Functional and biomimetic materials for engineering of the three-dimensional cell microenvironment. Chem. Rev..

[29] Ivanovic Z (2009). Hypoxia or in situ normoxia: the stem cell paradigm. J. Cell. Physiol..

[30] Boregowda SV (2012). Atmospheric oxygen inhibits growth and differentiation of marrow-derived mouse mesenchymal stem cells via a p53-dependent mechanism: implications for long-term culture expansion. Stem Cells.

[31] Drela K (2014). Low oxygen atmosphere facilitates proliferation and maintains undifferentiated state of umbilical cord mesenchymal stem cells in an hypoxia inducible factor-dependent manner. Cytotherapy.

[32] Kim H (2019). Mesenchymal stem cell 3D encapsulation technologies for biomimetic microenvironment in tissue regeneration. Stem Cell Res. Ther..

[33] Ventre M, Netti PA (2016). Controlling cell functions and fate with surfaces and hydrogels: the role of material features in cell adhesion and signal transduction. Gels.

[34] Francis KR, Wei L (2010). Human embryonic stem cell neural differentiation and enhanced cell survival promoted by hypoxic preconditioning. Cell Death Dis..

[35] Wang Y (2013). Hypoxia promotes dopaminergic differentiation of mesenchymal stem cells and shows benefits for transplantation in a rat model of Parkinson’s disease. PLoS ONE.

[36] Musah S (2014). Substratum-induced differentiation of human pluripotent stem cells reveals the coactivator YAP is a potent regulator of neuronal specification. PNAS.

[37] Skardal A, Mack D, Atala A, Soker S (2013). Substrate elasticity controls cell proliferation, surface marker expression and motile phenotype in amniotic fluid-derived stem cells. J. Mech. Behav. Biomed. Mater..

[38] Buzanska L (2010). Neural stem cells from human cord blood on bioengineered surfaces—novel approach to multiparameter bio-tests. Toxicology.

[39] Buzanska L, Zychowicz M, Sarnowska A, Domanska-Janik K (2013). Bioengineering of neural stem cell niche. Postepy. Biochem..

[40] Pennings S, Liu KJ, Qian H (2018). The stem cell niche: interactions between stem cells and their environment. Stem Cells Int..

[41] Robinson ST (2016). A novel platelet lysate hydrogel for endothelial cell and mesenchymal stem cell-directed neovascularization. Acta Biomater..

[42] Allen AB, Butts EB, Copland IB, Stevens HY, Guldberg RE (2017). Human platelet lysate supplementation of mesenchymal stromal cell delivery: issues of xenogenicity and species variability. J. Tissue Eng. Regen. Med..

[43] Chen B (2012). The effects of human platelet lysate on dental pulp stem cells derived from impacted human third molars. Biomaterials.

[44] Li H (2019). Immunomodulatory functions of mesenchymal stem cells in tissue engineering. Stem Cells Int..

[45] Kosinski M (2020). Bone defect repair using a bone substitute supported by mesenchymal stem cells derived from the umbilical cord. Stem Cells Int..

[46] Bianco P (2013). Regulation of stem cell therapies under attack in Europe: for whom the bell tolls. EMBO J..

[47] Martín-Martín Y (2019). Evaluation of neurosecretome from mesenchymal stem cells encapsulated in silk fibroin hydrogels. Sci. Rep..

[48] Sarnowska A (2009). Bilateral interaction between cord blood-derived human neural stem cells and organotypic rat hippocampal culture. Stem Cells Dev..

[49] Mastri M (2012). Activation of Toll-like receptor 3 amplifies mesenchymal stem cell trophic factors and enhances therapeutic potency. Am. J. Physiol. Cell Physiol..

[50] Chen Y, Shu Z, Qian K, Wang J, Zhu H (2019). Harnessing the properties of biomaterial to enhance the immunomodulation of mesenchymal stem cells. Tissue Eng. Part B Rev..

