# Supplementary material for: Biomimetic microenvironmental preconditioning enhance neuroprotective properties of human mesenchymal stem cells derived from Wharton's Jelly (WJ-MSCs)
Source: Sci Rep. 2020 Oct 9;10:16946. doi: 10.1038/s41598-020-74066-0 (PMC7547118; doi:10.1038/s41598-020-74066-0)
Supplement: Supplementary file 1 — Supplementary file1 [file 41598_2020_74066_MOESM1_ESM.pdf]

Biomimetic microenvironmental preconditioning enhance neuroprotective properties of human mesenchymal stem cells derived from Wharton's Jelly (WJ-MSCs)

Wioletta Lech[1], Anna Sarnowska[1,2], Zuzanna Kuczynska[1], Filip Dabrowski[3], Anna Figiel-Dabrowska[2], Krystyna Domanska-Janik[1], Leonora Buzanska[1], Marzena Zychowicz[1]*

Supplementary materials

Supplementary table 1. Primary antibodies used for immunocytochemistry.

Primary antibody	Source	Isotype	Dilution	Company
Nestin	mouse monoclonal	IgG1	1:200	Millipore
βTubulinIII	mouse monoclonal	IgG2b	1:1000	Sigma-Aldrich
NF-200	mouse monoclonal	IgG1	1:400	Sigma-Aldrich
GFAP	rabbit polyclonal	IgG (H+L)	1:500	Dako

Supplementary table 2. Primers used for quantitative RT-PCR.

Gene	Product size	Primersequence (5'→3')
ACTB	120 bp	F: GCCAACCGCGAGAAGATGA<br>R: CATCACGATGCCAGTGGTA
GDNF	92 bp	F: TTTAGGTACTGCAGCGGCTCTT<br>R: TCACTCACCAGCCTTCTATTTCTG
BDNF	80 bp	F: ATTACAATCAGATGGGCCACATG<br>R: AGGGAGAAAGCAGAAACAAGACA
EGF	85 bp	F: GCAGAGGGATACGCCCTAAGT<br>R: CAAGAGTACAGCCATGATTCCAAA
bFGF	123 bp	F: CAAGCGGCTGTACTGCAAAA<br>R: CCTCTCTCTTCTGCTTGAAGTTGTA
VEGF-A	91 bp	F: ATGACGAGGGCCTGGAGTGTG<br>R: CCTATGTGCTGGCCTTGGTGAG
TGF-β1	101 bp	F: CCCAGCATCTGCAAAGCTC<br>R: GTCAATGTACAGCTGCCGCA
IL-6	81 bp	F: GGTACATCCTCGACGGCATCT<br>R: GTGCCTCTTTGCTGCTTTCAC
IL-1β	129 bp	F: GGGACAGGATATGGAGCAACA<br>R: TCTTTCAACACGCAGGACAG
Nestin	169 bp	F: TGGCTCAGAGGAAGAGTCTGA<br>R: TCCCCCATTTACATGCTGTGA
βTubulinIII	159 bp	F: CTCAGGGGCCTTGGACATC<br>R: CAGGCAGTCGCAGTTTTCAC
NF-200	160 bp	F: GAGGAACACCAAGTGGGAGA<br>R: TTCTGGAAGCGAGAAAGGAA
GFAP	266 bp	F: GCAGAGATGATGGAGCTCAATGACC<br>R: GTTTCATCCTGGAGCTTCTGCCTCA
